# Recombinant HIV Envelope Proteins Fail to Engage Germline Versions of Anti-CD4bs bNAbs

**DOI:** 10.1371/journal.ppat.1003106

**Published:** 2013-01-03

**Authors:** Sam Hoot, Andrew T. McGuire, Kristen W. Cohen, Roland K. Strong, Lars Hangartner, Florian Klein, Ron Diskin, Johannes F. Scheid, D. Noah Sather, Dennis R. Burton, Leonidas Stamatatos

**Affiliations:** 1 Seattle Biomedical Research Institute, Seattle, Washington, United States of America; 2 University of Washington, Department of Global Health, Seattle, Washington, United States of America; 3 Fred Hutchinson Cancer Research Center, Seattle, Washington, United States of America; 4 Department of Immunology, IAVI Neutralizing Antibody Center and Center for HIV/AIDS Vaccine Immunology and Immunogen Discovery, The Scripps Research Institute, La Jolla, California, United States of America; 5 Laboratory of Molecular Immunology, The Rockefeller University, New York, New York, United States of America; 6 Division of Biology, California Institute of Technology, Pasadena, California, United States of America; 7 Ragon Institute of MGH, MIT, and Harvard, Cambridge, Massachusetts, United States of America; SAIC-Frederick, United States of America

## Abstract

Vaccine candidates for HIV-1 so far have not been able to elicit broadly neutralizing antibodies (bNAbs) although they express the epitopes recognized by bNAbs to the HIV envelope glycoprotein (Env). To understand whether and how Env immunogens interact with the predicted germline versions of known bNAbs, we screened a large panel (N:56) of recombinant Envs (from clades A, B and C) for binding to the germline predecessors of the broadly neutralizing anti-CD4 binding site antibodies b12, NIH45-46 and 3BNC60. Although the mature antibodies reacted with diverse Envs, the corresponding germline antibodies did not display Env-reactivity. Experiments conducted with engineered chimeric antibodies combining the mature and germline heavy and light chains, respectively and vice-versa, revealed that both antibody chains are important for the known cross-reactivity of these antibodies. Our results also indicate that in order for b12 to display its broad cross-reactivity, multiple somatic mutations within its VH region are required. A consequence of the failure of the germline b12 to bind recombinant soluble Env is that Env-induced B-cell activation through the germline b12 BCR does not take place. Our study provides a new explanation for the difficulties in eliciting bNAbs with recombinant soluble Env immunogens. Our study also highlights the need for intense efforts to identify rare naturally occurring or engineered Envs that may engage the germline BCR versions of bNAbs.

## Author Summary

Recombinant HIV Envelope glycoproteins (Env), the sole target of anti-HIV neutralizing antibodies, have, so far, not been able to elicit broadly neutralizing antibodies (bNAbs) although they express the corresponding epitopes. Such constructs elicit neutralizing antibodies of very narrow neutralizing breadth; antibodies whose epitopes are primarily located within variable domains of Env. Diverse approaches that have been evaluated over the past two decades to overcome this limitation were met with limited success. The exact reasons for the lack of elicitation of bNAbs during immunization with Env are not well understood. Here we show that recombinant Env proteins are inefficient in engaging the predicted germline BCRs of known bnAbs. Thus, our study provides new insights as to why recombinant Env immunogens have failed to elicit bNAbs. Our results indicate that, as a first step in eliciting bNAbs by immunization, Env immunogens should be designed that would engage the germline BCR versions of bNAbs.

## Introduction

Broadly neutralizing anti-HIV antibody responses are generated by approximately 15% of those infected with HIV-1 [1]–[4] and monoclonal antibodies (MAbs) with broad and potent anti-HIV neutralizing activities have been isolated from chronically HIV-1-infected subjects [5]–[11]. The structures and locations on the viral envelope glycoprotein (Env; the sole target of anti-HIV neutralizing antibodies) of the epitopes recognized by many broadly neutralizing MAbs have been characterized [6], [7], [12]–[15] and recombinant forms of Env have been engineered to express the epitopes of broadly neutralizing MAbs. Such Env proteins have been shown to be recognized by known broadly neutralizing MAbs and to adsorb the broadly neutralizing activities of HIV+ sera [2], [3], [16]–[22]; indications that these proteins effectively present the epitopes targeted by bNAbs generated during HIV-infection. Despite however, the presence of epitopes recognized by bNAbs on such Env proteins, these constructs when used as immunogens fail to elicit similar types of broadly neutralizing antibody responses [16], [23]–[35]. To overcome this major obstacle in HIV vaccine-development, diverse approaches were evaluated over the past two decades, (reviewed in [36]). So far however, these approaches were met with very limited success.

The recent characterization of several broadly neutralizing MAbs against the CD4-BS of Env revealed that the VH and VL regions of these antibodies can be up to 50% divergent from the corresponding germline sequences [7], [37]. Xiao *et al.* reported that the scFv form of the germline version of the broadly neutralizing anti-CD4-BS antibody b12 does not bind clade B recombinant Env proteins 89.6, Bal, JRFL and R2 [38], [39]. Scheid *et al*., reported that the germline versions of certain broadly neutralizing anti-CD4-BS MAbs fail to bind the Envs that were used to isolate the mature version of these antibodies [7]. These observations were made with a very limited number of recombinant Env proteins and thus far, the engagement of the germline BCR versions of bNAbs by recombinant HIV Env immunogens has not been examined in any great extent.

To compare the interactions of recombinant Env immunogens with the germline and mature forms of bNAbs we focused on well-characterized anti-HIV neutralizing antibodies whose epitopes are located in the CD4-BS of Env; namely MAbs b12, NIH45-46 and 3BNC60. These MAbs were isolated from 3 different HIV-infected subjects [5], [7]. All three antibodies can neutralize a wide range of primary viruses from distinct clades, although the breadth and potency of NIH45-46 and 3BNC60 are far superior to those of b12 [7], [11]. Passive infusion studies conducted in non-human primates with b12 have indicated that this antibody can protect animals from experimental SHIV-infection [40].

The recombinant Envs used in our studies were derived from diverse isolates from clades A, B and C (the three most predominant clades worldwide [41], [42]) and include constructs that have been already tested in preclinical and clinical studies, including the recent RV144 clinical trial [43]. We investigated the interactions between soluble recombinant Env and the mature and germline versions of these antibodies. We additionally sought to characterize the relative contributions of the mature and germline heavy and light chains to Env recognition, and to characterize how affinity maturation at key residues within the b12 heavy chain affects recognition of Env. Our results provide new insights into the interaction between HIV Env immunogens and bNAbs to the CD4-BS and provide alternative explanations as to why soluble Env immunogens have so far failed to elicit broadly neutralizing antibody responses.

## Results

### Mature and germline b12 VH and VL sequence comparison

The b12 germline V, D and J genes were sequenced from the subject from which b12 was isolated [44]. The gene usage of b12 is VH1-03*01, D2-21*02 and J6*03 for VH, and KV3-20*01 and KJ2*01 for VL. The VH and VL gene-usage for b12 differs from that of several recently identified bNAbs that also target epitopes within the CD4-BS of Env, such as VRC01, NIH45-46 or 3BNC60 [7], [37]. The gene usage of these antibodies is VH1-2 for all three, and KV3-11 for VRC01 and NIH45-46, and KV1D-33 for 3BNC60.

**Figure 1:**
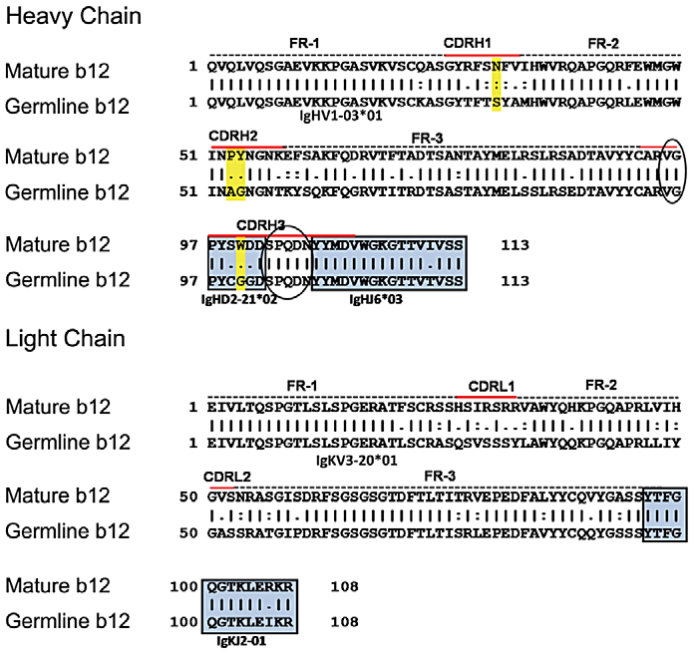
Amino acid alignment of b12 mature and germline heavy and light chain variable regions. The framework (FR) and complementary determining regions (CDR) are outlined, and the D- and J- gene segments are boxed. The complementarity determining (CDR) and framework (FW) regions were determined using the IMGT/V-Quest tool (www.imgt.org). Amino acid numbering is based on the Kabat numbering system. Seven amino acids (two in the VD joining region and five in the DJ joining region) that are present in the mature sequence (and which were left unchanged in the germline sequence used here) are shown in oval. The four amino acids in the VH chain that are known to make direct contact with the gp120 core are highlighted in yellow.

In the b12 mature heavy chain sequence, there are seven amino acid residues flanking the predicted D gene segment; two (V95 and G96) in the VD junction and five (S103, P104, Q105, D106 and N107) in the DJ junction (**Figure 1**). Because it is not possible to predict if the seven amino acid insertions took place during the VD and DJ recombinations, or if they were introduced during B cell affinity maturation, these seven amino acids were left unchanged while constructing the b12 germline VH sequence used herein. The actual b12 germline VH chain differs from the previously published predicted b12 germline [39] at two positions: C99 (reported as S99 previously) and G101 (reported as D101 previously). The b12 mature and germline VH sequences differ by 21% (24 amino acids) and the mature and germline VL sequences differ by 19% (21 amino acids).

The mature NIH45-46 and 3BNC60 VH and VL regions have acquired a higher number of somatic mutations (**Figure S1**). As a result, NIH45-46 VH mature and germline sequences differ by 39% (44 amino acids), while the VL mature and germline sequences differ by 25% (27 amino acids). The 3BNC60 germline and mature VH sequences differ by 39% (46 amino acids), while the VL mature and germline sequences differ by 30% (29 amino acids).

### The soluble germline version of IgG b12 does not bind recombinant HIV-1 envelopes from a diverse panel of viruses

**Figure 2:**
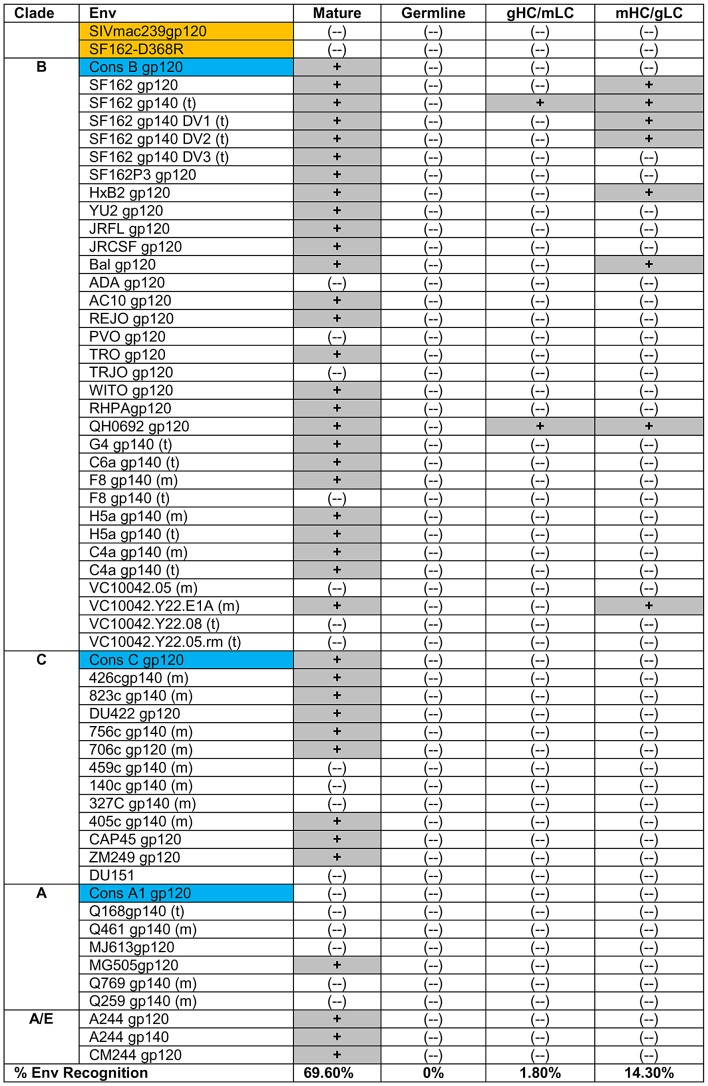
Binding of mature, germline and chimeric IgG b12 forms to the indicated recombinant Env was determined by ELISA as described in the **Materials and Methods** **section.** (−): no binding; (+): binding; gp140(t): trimeric gp140; gp140(m): monomeric gp140.

Xiao *et al.* reported that the germline version of IgG b12 did not interact with recombinant gp120 derived from the clade B viruses Bal, JRFL, 89.6 and R2 [38]. Here we examined whether the germline IgG b12 could interact with any of the recombinant Envs in a large panel of Envs derived from clade A, B and C viruses (**Figure 2**). These Envs included monomeric gp120 forms, as well as monomeric and trimeric gp140 forms. Many of these proteins have been previously evaluated as immunogens to elicit bNAbs in pre-clinical and clinical studies.

By ELISA, mature IgG b12 bound to 39 of 56 Envs tested (∼69%). As expected, binding to the SF162-D368R gp120 or SIVmac239 gp120, was not observed. The germline IgG b12 did not display detectable binding to any of the envelopes tested, irrespective of the clade of Env, whether it was in monomeric gp120 or trimeric gp140 form, and whether the variable regions 1, 2 or 3 (SF162ΔV1, SF162ΔV2 and SF162ΔV3, respectively) were present or not.

**Figure 3:**
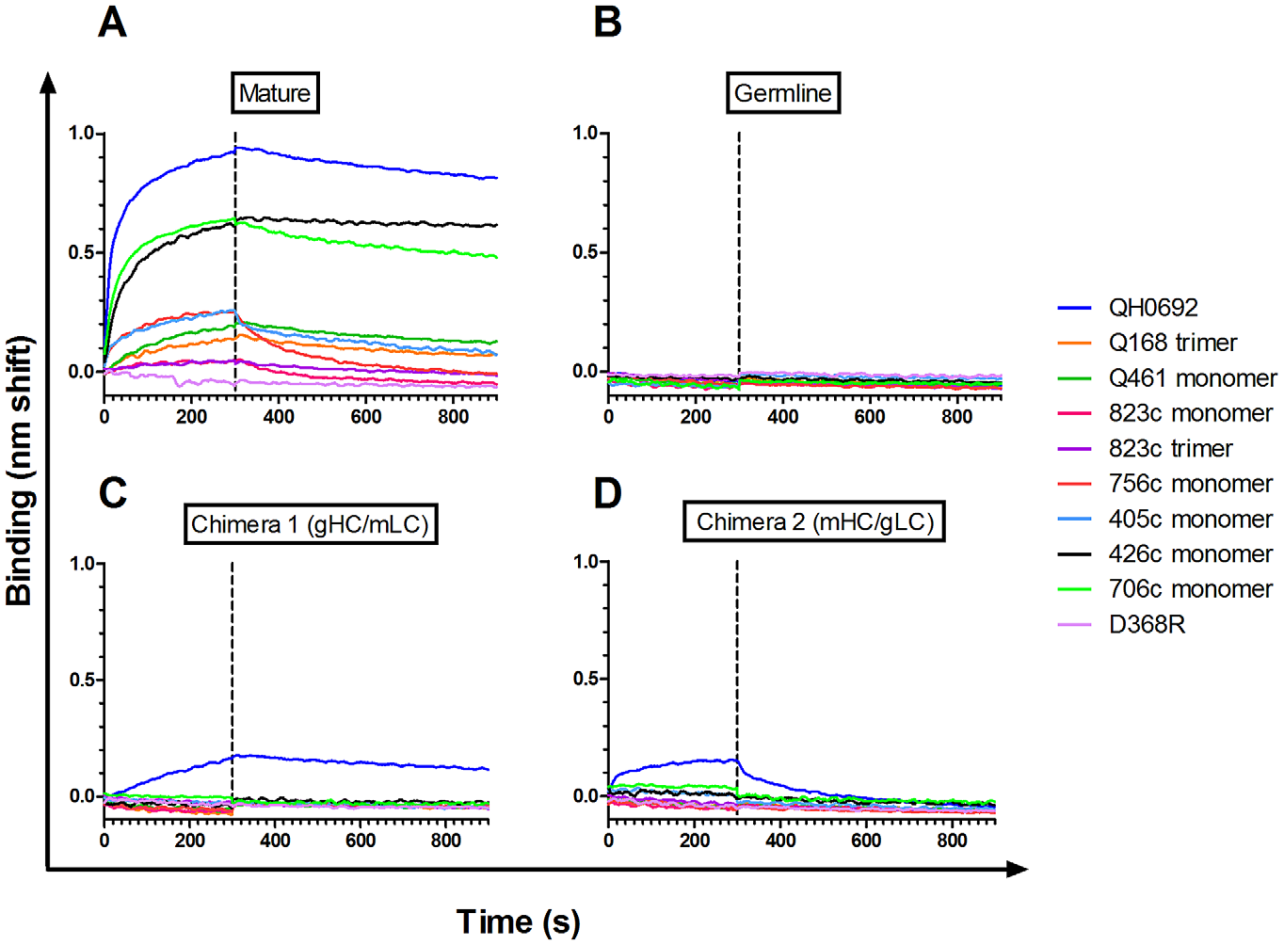
Binding of mature, germline and mature/germline b12 chimeras to Env. Mature (**A**), germline (**B**), chimera 1 (**C**), or chimera 2 (**D**), IgGs were immobilized to anti-human IgG FC capture BLI biosensors and Env proteins were in solution at 1.2 µM. The QH0692 and D368R Envs were in gp120 form. The remaining Envs were in gp140 form (either as trimers or as monomers).

In parallel to the ELISA-based screening, we performed Biolayer Interferometry (BLI) analysis to characterize in real-time, any weak and transient interactions of the mature and germline b12 with a subset (ten) of Envs (**Figure 3**). Mature b12 binding was detectable to all of the Envs that showed detectable b12-binding by ELISA (QH0692, 756c, 405c, 426c and 706c), as well as two Envs that did not show ELISA binding, Q168a2 (orange) and Q461e2 (green) (**Figure 3A**). In contrast, and in complete agreement with the ELISA results, no interaction between the germline b12 and any of the Envs tested was observed (**Figure 3B**).

### Both heavy and light chains of b12 are important for binding to Env

The crystal structure of mature b12 in complex with the HIV-1 HxB2 gp120 core revealed that b12 binds Env exclusively through its heavy chain [15]. Zwick *et al*. however reported that artificially introduced mutations within the variable regions of the light chain of b12, can dramatically affect the binding of the mature IgG b12 to Env [45].

To investigate in more detail the relative contributions of the heavy and light chains of b12 to its ability to recognize Env, we produced recombinant b12 chimeric IgGs containing either the germline heavy chain paired with the mature light chain (Chimera 1, gHC/mLC) or the mature heavy chain paired with the germline light chain (Chimera 2, mHC/gLC) (**Figure S2**). We screened the gHC/mLC and mHC/gLC IgG chimeras for binding by ELISA and/or BLI to the panel of Envs listed in **Figure 2** **and** **Figure 3**. The gHC/mLC (Chimera 1) bound consistently to a single Env; derived from the Clade B virus QH0692 (**Figure 2** **and** **Figure 3C**).

**Figure 4:**
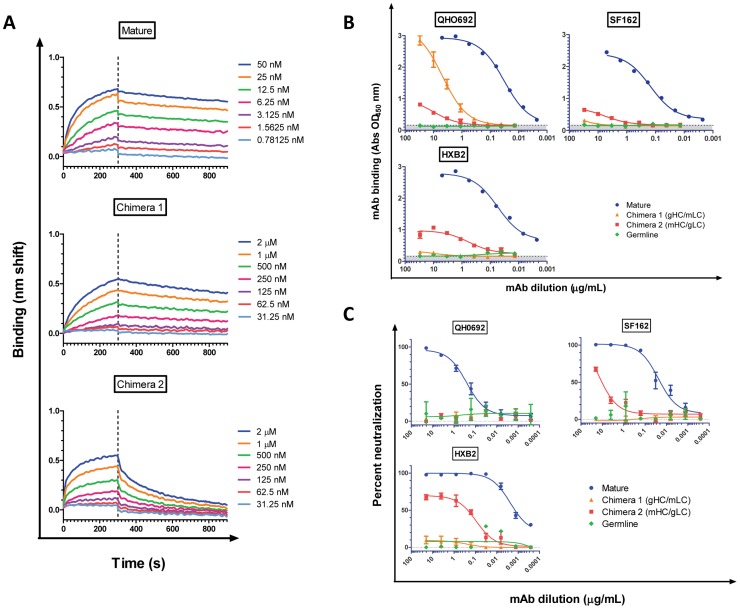
Binding and neutralizing properties of the b12 mature, germline and mature/germline b12 chimeras to Env. (**A**) Kinetic analysis of binding of mature b12 and of the two chimeras to QH0692 gp120. The Env concentrations tested are shown. A summary of the results is presented in **Table 1**. (**B**) IgG-binding to QH0692, SF162 and HXB2 gp120s by ELISA. The shaded grey area indicates background (non-specific) binding (determined as described in the Materials and Methods section). All binding curves are representative from two to four independent experiments. (**C**) Neutralizing activities of IgG against QH0692, SF162 and HXB2 virions. Results are representative from two to four independent experiments.

In contrast to the rare Env-reactivity displayed by the gHC/mLC chimera, the mHC/gLC chimera (Chimera 2) displayed detectable binding not only to QH0692, but also to three additional clade B Envs (SF162, HXB2 and VC10042.Y22.E1A) by ELISA (**Figure 2** **and** **Figure 4B**). However, the mHC/gLC chimera did not interact with any of the non-clade B Envs tested. We note that the presence or absence of the first or second hypervariable regions of gp120 (V1 and V2 loops respectively) did not affect the recognition of the SF162 gp140 by chimera mHC/gLC. Interestingly however, there was no detectable binding to the SF162 Env once the V3 loop was deleted.

Collectively this analysis highlights the importance of the b12 heavy chain as the primary recognition determinant of diverse Envs, but also shows that the light chain plays an important role in the ability of b12 to recognize diverse Envs.

To further characterize the interactions of gHC/mLC and mHC/gLC with QH0692 Env (which was the only Env from the panel tested here that consistently bound to the mature and both chimeric forms of b12), we performed binding kinetic analysis by BLI (**Figure 4A**). The results from this analysis are summarized in **Table 1**. The binding affinities (K_a_) for both chimeras were lower (by 2–3 Log_10_) than for the mature b12, which had an affinity of 2.0 nM for QH0692 gp120. The lower affinity of the gHC/mLC compared to mature b12 is due to a 2 Log_10_ slower association rate (*k*_a_). The mHC/gLC lower *K*_a_ compared to mature b12 is primarily due to a faster dissociation rate (*k*_d_), although we note that the *k*_a_ of the mHC/gLC chimera is slower as well.

**Table 1:** Kinetic parameters of the mature and chimeric IgG b12 forms for the QH0692 gp120.

	b12	std error	gHC/mLC	std error	mHC/gLC	std error
*K*_a_ (M^−1^)	2.00E+09	3.16E+08	1.39E+07	3.20E+06	7.56E+06	1.37E+06
*k*_a_	7.87E+05	2.19E+05	7.76E+03	1.97E+03	4.85E+04	1.21E+04
*k*_d_	4.01E-04	4.14E-05	5.48E-04	1.28E-06	5.97E-03	5.31E-04

### mHC/gLC but not gHC/mLC b12 IgG neutralizes certain HIV-1 viruses

To determine whether the binding of b12 gHC/mLC and mHC/gLC to certain soluble Envs translates into binding to the corresponding virion-associated Env and leads to inhibition of viral infectivity, we compared their abilities to bind Env from QH0692, SF162 and HXB2 (**Figure 4B**) and to neutralize the corresponding virions (**Figure 4C**). The gHC/mLC only bound QH0692 Env, while the mHC/gLC bound all three Envs but with different affinities. Interestingly, while mHC/gLC bound much less efficiently to QH0692 than gHC/mLC, it bound with higher affinity to SF162 and HxB2 gp120s, while gHC/mLC did not recognize these Envs.

Mature b12 IgG neutralized all three viruses, with IC_50_ values of 0.22 µg/mL for QH0692, 0.01 µg/mL for SF162, and 0.002 µg/mL for HXB2. The mHC/gLC chimera did not neutralize QH0692, but was able to neutralize HxB2 with an IC_50_ value of 4.0 µg/mL and also neutralized SF162, but significantly less potently. The observation that the neutralizing potency of mHC/gLC against SF162 and HxB2 was reduced as compared to the neutralizing potency of mature b12, further supports the important role that that the light chain plays in the binding and neutralizing activities of b12. The observed higher neutralization potency of mHC/gLC against HXB2 than SF162 is most likely due to the increased binding affinity of mHC/gLC to HXB2 Env (**Figure 4B**).

Despite being able to bind the recombinant QH0692 Env, gHC/mLC and mHC/gLC were unable to neutralize the QH0692 virus, even at the highest concentration of mAb tested (20 µg/mL). Potentially, this lack of neutralizing activity could be due to a slower *k*_a_ and/or faster *k*_d_ of the two chimeric IgGs as compared to those of the mature b12 (**Figure 4A** **and** **Table 1**).

### Interaction of mature and germline versions of NIH45-46 and 3BNC60 with recombinant HIV Env

**Figure 5:**
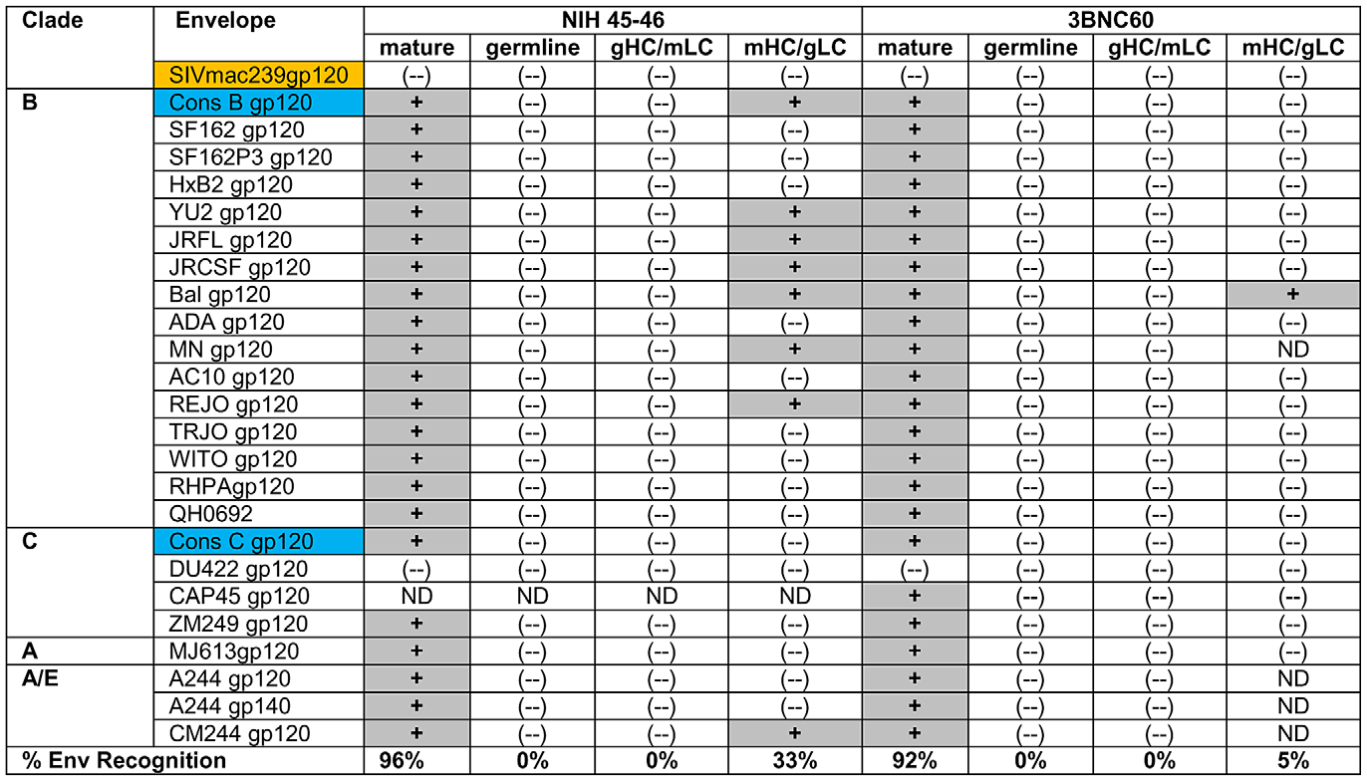
Binding of mature, germline and chimeric IgG NIH45-46 and 3BNC60 to Env. Antibody binding to each Env was determined with BLI using the antigen capture method as described in the Materials and Methods section. Symbols as in Figure 2.

b12 was isolated by phage display [46], [47] and the light chain used to create the mature b12 form may not have been naturally paired with the b12 heavy chain. Thus, the above described results on the importance of the light chain of b12 in the interaction of the mature and germline forms with Env can simply be the result of improperly matched heavy and light chains. To investigate whether the binding results discussed above between Env and the mature/germline heavy/light chain chimeric b12 IgGs, are specific to b12, we characterized the relative contributions of mature and germline heavy and light chains to Env binding for other CD4-BS antibodies. Recently, several anti-CD4-BS antibodies with broad and potent neutralizing activities have been isolated from HIV-infected subjects [7], [9]. Since these antibodies were isolated from single sorted B cells, the heavy and light chains are naturally occurring cognate pairs. Two such antibodies are NIH45-46 and 3BNC60 [7], [11]. We produced recombinant mature, germline, gHC/mLC and mHC/gLC chimeric versions of NIH45-46 and 3BNC60 and investigated their interactions with a panel of recombinant gp120s from diverse genetic clades (**Figure 5**) using BLI . Mature NIH45-46 bound to 23 of 24 HIV gp120s tested while mature 3BNC60 bound to 19 of 20 HIV gp120s tested. Binding of germline NIH45-46 or gHC/mLC NIH45-46 to any of the Envs tested was undetectable. In contrast, the NIH 45-46 mHC/gLC bound to 8 of the 24 Envs tested. The mature 3BNC60 bound to 23 of 25 Envs tested while mHC/gLC was able to bind to a single Env tested, that from BAL. The gHC/mLC chimera did not bind to any Envs tested.

Combined, our results indicate that both the heavy and light chains of anti-CD4-BS bNAbs play important roles in Env binding, but that binding to some variants is maintained when either the heavy or light chains are substituted for the corresponding germline sequences.

### Soluble Env trimers stimulate B-cell activation through the mature but not through the germline b12 BCR

**Figure 6:**
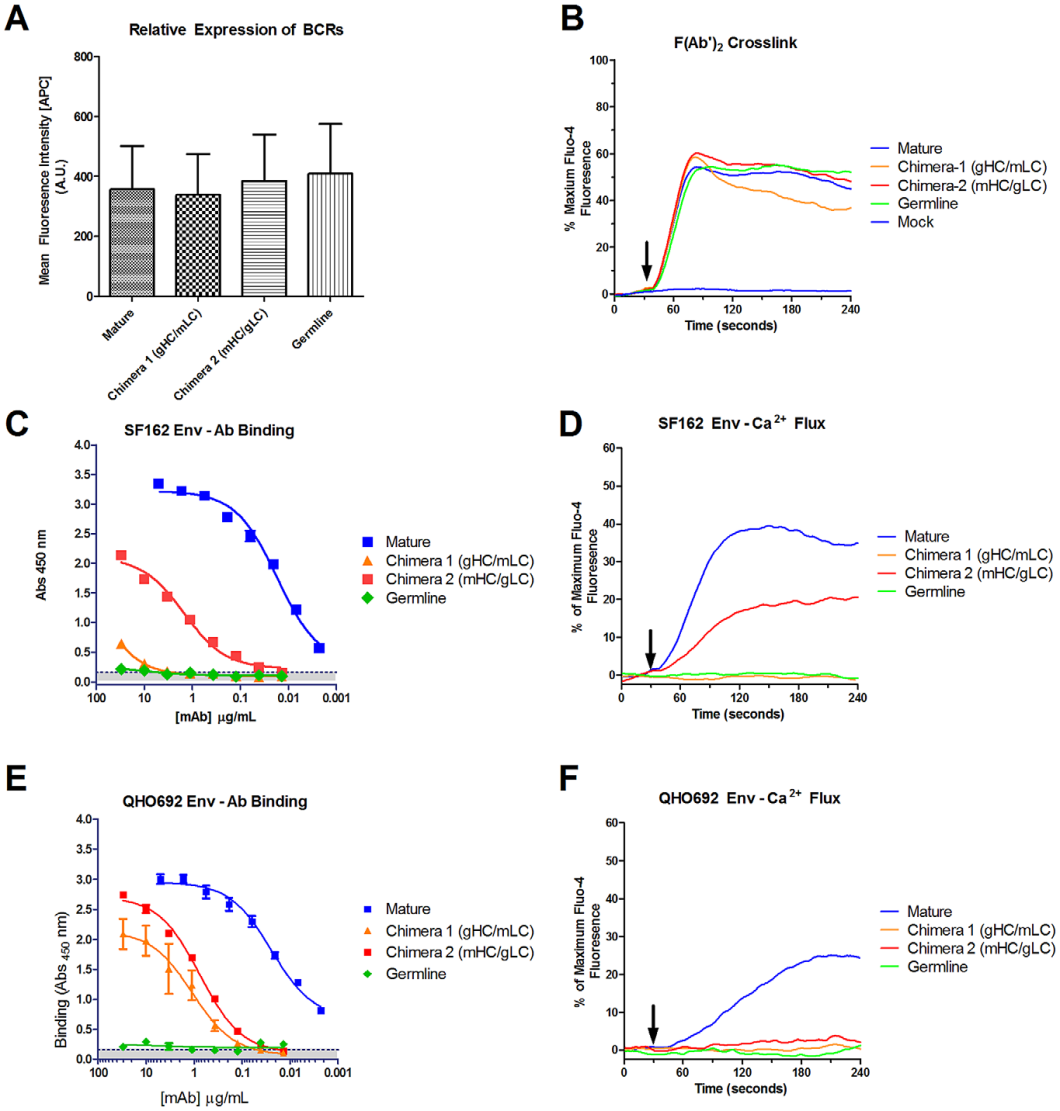
Env-specific B-cell activation through mature and chimeric b12 BCRs. (**A**) Cell-surface expression of b12 mature, germline and chimeric BCRs on the surface of B cells. (**B**) Intracellular Ca^2+^ flux mediated by the mature, germline and chimeric BCRs following BCRs cross-linking by goat anti-human IgG (H+L) F(ab′)_2_ in A20 cells. (**C**) Binding of mature, germline and chimeras to SF162 gp140 trimer. (**D**) Intracellular Ca^2+^ flux upon addition of SF162 gp140 trimers to B cells (DG-75) expressing the indicated b12 BCRs. (**E**) Binding of mature, germline and chimeras to QH0692 gp140 trimer. (**F**) Intracellular Ca^2+^ flux upon addition of QH0692 gp140 trimers to B cells expressing the indicated b12 BCRs.

The above discussed binding experiments were performed with soluble versions of the mature and germline b12 IgG. In the context of B cell stimulation by an antigen however, the BCR is anchored on the plasma membrane. Upon binding of antigen to the cell surface-expressed BCRs and following BCR cross-linking, specific intracellular activation signaling takes place. One consequence of B cell activation is intracellular calcium mobilization. It is possible that very weak interactions of BCRs with Env immunogens can lead to B cell activation, even though these interactions are either too weak to be determined biophysically, or because these interactions are not taking place when the soluble version of BCRs are used. To determine whether recombinant Env can induce B-cell activation through the b12 BCR, we monitored intracellular calcium mobilization of B cells expressing either the mature, germline, or the above discussed chimeric b12 BCRs in response to SF162 gp140 trimer and QH0692 gp140 trimer. All four BCRs were expressed on the cell surface at similar levels (**Figure 6A**) and all four were functional as indicated by Ca^2+^ flux when the BCRs were cross-linked on the surface of B cells by anti-human IgG F(ab′)2 antibodies (**Figure 6B**).The mature b12 bound to the SF162 gp140 trimers with greater affinity than the mHC/gLC chimera, as observed with SF162 gp120 (**Figure 4B**), although the binding to trimeric gp140 was more efficient than that for gp120 (compare **Figure 4B** with **Figure 6C**). Very weak binding (detectable only at the highest IgG concentrations used and not in every independent experiment) was recorded with the gHC/mLC chimera while no binding was detectable by the germline b12 (**Figure 6C**). The degree of calcium mobilization upon addition of SF162 gp140 trimer correlated with the strength of Env-IgG binding recorded by ELISA. Robust Ca^2+^ flux was observed in response to mature b12 BCR engagement, an intermediate level of flux in response to mHC/gLC BCR engagement, and no Ca^2+^ flux via the gHC/mLC or germline BCRs (**Figure 6D**). These results are in agreement with reports that the magnitude and duration of BCR signaling is critically dependent on the affinity of the BCR-antigen interaction [48].

The QH0692 gp140 trimer bound with high affinity to mature b12 and to both chimeras (**Figure 6E**). We note that the binding of gHC/mLC to the trimeric gp140 was far superior to the binding to gp120 (compare **Figure 4B** with **Figure 6E**). However, calcium mobilization was observed only through the mature b12 BCR (**Figure 6F**). Potentially, the orientation of the b12 epitope on the QH0692 gp140 trimers is such that it is not conducive to BCR cross-linking on the B cell surface.

### Germline-to-mature mutations acquired during affinity maturation confer increased Env-binding affinity and HIV neutralization potency

**Figure 7:**
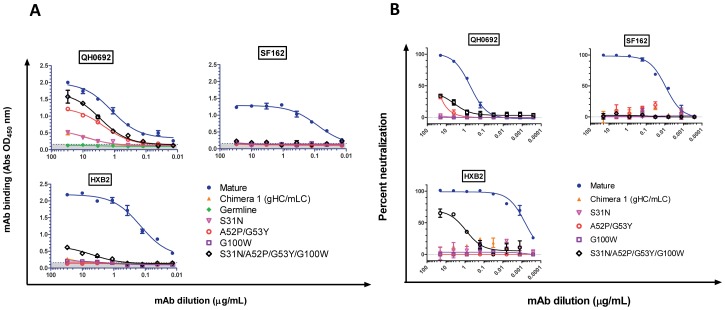
Effect of germline-to mature VH mutations on the binding and neutralizing activity of IgG b12 expressing the mature VL chain. (**A**) The S31, A52P and G53Y, G100W, and S31N/A52P/G53Y/G100W amino acid mutations were introduced on the germline VH chain. The mutated heavy chains were combined with the mature light chains and the corresponding IgGs were made. Their binding to the indicated recombinant Envs was determined. The binding of the fully mature b12, that of the germline b12 and that of the gHC/mLC chimera were also determined. The shaded grey area indicates background (non-specific) binding (determined as described in the Materials and Methods section). All binding curves are representative from two to four independent experiments. (**B**) Neutralizing activities of the same IgGs against the indicated virions.

Within the heavy chain of mature b12, four amino acids make up 40% of the direct contact surface with Env [15]. These four amino acids are N31 (in CDRH1), P52 and Y53 (in CDRH2) and W100 (in CDRH3) (highlighted in yellow in **Figure 1**). In the germline sequence these four positions are occupied by different amino acids: S31, A52, G53 and G100. These amino acid changes occurred therefore by AID-mediated somatic hypermutation during affinity maturation of Env-specific B cell clones in the subject from which b12 was isolated. To better understand how germline-to-mature mutations in these four positions affect the ability of b12 to recognize and neutralize diverse viruses, we substituted the amino acids found in the germline sequence to those found in the mature sequence. The mutations introduced were: S31N, A52P and G53Y, G100W, and S31N/A52P/G53Y/G100W (quadruple mutant). The germline heavy chain with these mutations was co-expressed with either the mature light chain or the germline light chains and the IgGs were tested for their binding and neutralization properties (**Figure 7**).

When paired with the germline light chain, none of these mutations conferred Env-binding or neutralization (data not shown). When paired with the mature light chain, the effect of these germline-to-mature mutations on Env-binding and HIV-neutralization was Env and virus target-dependent. None of these mutations conferred binding to SF162 Env (**Figure 7A**) or SF162 neutralization (**Figure 7B**). In the case of the SF162 Env therefore, additional germline-to-mature mutations are required for binding and neutralization. The quadruple mutant bound the HXB2 Env and neutralized HXB2, albeit at a ∼4× Log_10_ reduction compared to mature b12. The S31N, A52P/G53Y, and G100W mutants bound only to the QH0692 Env, as did the quadruple mutant. Interestingly, binding of these variants to recombinant QH0692 Env did not result in QH0692 virus neutralization.

## Discussion

The epitope of b12 is expressed on numerous recombinant Env-derived immunogens that have been tested pre-clinically and clinically for the elicitation of antibody responses similar to b12, but there is no indication that b12-like neutralizing antibodies have been elicited by such constructs. The reasons for the failure of recombinant Env immunogens to elicit b12-like neutralizing activities are not well understood. Understanding therefore how b12 was generated in the context of natural infection is a critical piece of information that is currently missing; information that would be very useful for the design of immunogens capable of eliciting b12-like antibodies.

Our results indicate that the germline version of b12 is not recognized by diverse Envs from clades A, B or C; Envs which are recognized by the mature b12 antibody. Our results therefore suggest that a potential reason why recombinant soluble Env immunogens have failed so far to elicit b12-like antibody responses may be the inability of these constructs to engage the germline BCR versions of such antibodies. In the absence of such an engagement, the process of B cell maturation that would lead to the eventual production of b12-like antibody is unlikely to be initiated.

The variable antibody regions of the mature and germline sequences of b12 differ by 21% for the heavy chain and 19% for the light chain and even more importantly, the four VH amino acids of mature b12 that make direct contact with Env, differ between the mature and germline sequences. When these four key amino acids are mutated (individually or in combination) into those present in the mature VH sequence (germline-to-mature mutations) the germline heavy chain acquires Env-binding abilities. However, the binding is dependent on the co-expression of a mature variable light chain and is Env target dependent. In fact, in certain cases, germline-to-mature conversion of all four key positions in the VH chain does not lead to recognition of certain Envs that are readily recognized by the fully matured antibody. These observations suggest that the broad Env-recognition ability of b12 necessitates a larger number of somatic mutations to take place in the VH region during affinity maturation. Also, germline-to-mature conversion of all four positions did not always lead to HIV-neutralization even when they allowed Env-binding. Therefore the broad neutralizing activity of b12 was acquired following the introduction of multiple mutations in the germline VH region. Some of these mutations may have allowed the maturing b12-like antibody to bind Env without displaying its broad neutralizing potential. Identifying the minimal number of germline-to-mature mutations that are necessary for b12-like antibodies to display cross-neutralizing activities will assist the design of immunogens and immunization protocols that will lead to the minimum maturation required of the germline b12 VH sequence to adopt its broadly neutralizing form.

The critical role of the heavy chain of b12 for Env-binding is highlighted by the fact that when the mature heavy chain is substituted by the germline heavy chain (while the light chain is kept in its mature form) only a single recombinant Env (out of 53 tested) was recognized. This Env, QH0692, is derived from a clade B virus isolated during the first 3 months of infection from a patient from Trinidad [49]. If only the heavy chain, and not the light chain of b12 interacts with Env (as revealed by the crystal structure of b12 bound to the HxB2 gp120 core [15], then it is possible that the germline heavy chain of b12 binds the QH0692 Env. In this regard, the QH0692 Env is unique (for currently unknown reasons) among all the recombinant Envs tested here.

The identification of a recombinant Env that is recognized by the germline heavy chain of b12 supports the hypothesis that a very small number of Envs are capable of engaging the germline BCRs of b12-like antibodies. Also, this finding supports the utilization of QH0692-derived constructs as future immunogens to engage the fully germline version of the b12 BCR. Interestingly, the QH0692 Env was not recognized by the gHC/mLC of the other two anti-CD4-BS antibodies studied here. In fact the gHC/mLC versions of NIH45-46 and of 3BNC60 did not recognize any of the Envs tested. These observations suggest that a single Env might not engage the germline BCR of these three antibodies. From the point of view of immunogen design then, more than one Env immunogen may need to be engineered to engage all three germline BCRs tested here.

Reversion of the mature light chain to germline light chain abrogates the ability of the antibody to bind the majority of Envs. Thus, somatic mutations in the germline light chain are required to take place for the most optimal orientation of the heavy chain for Env-binding, or for stabilizing the interaction of the heavy chain with Env. The optimal engagement of b12-like antibodies with Env appears therefore to require multiple somatic mutations to take place in both light and heavy chains. Similar observations were made with two additional anti-CD4-BS antibodies, NIH45-46 and 3BNC60. Overall our results support the hypothesis that the pathway of affinity maturation which is required for the binding of anti-CD4-BS antibodies to diverse HIV Envs depends on somatic mutations introduced within both the heavy and light chains of these antibodies. Our data also suggests that depending on the Env target, the relative contribution of the heavy and light chains to Env recognition will vary.

Our results also indicate that the ability to bind diverse Envs is acquired gradually during affinity maturation, and is not inherent in the germline versions of the antibodies studied here. Importantly, the above described results show that some Envs can bind partial germline antibodies. Such Envs can serve as platforms to design Env proteins capable of engaging the germline versions of the antibodies examined here.

Although the above observations were made with the soluble versions of the mature, germline and chimeric antibodies, they are biologically relevant. The germline b12 BCR was unable to mediate intracellular calcium mobilization (an indication of B cell activation via BCR-antigen engagement). When the germline heavy chain was combined with the mature light chain, Env-mediated B cell activation was not observed. In contrast when the mature heavy chain was combined with the germline light chain, Env-specific B cell activation was observed, but at levels far reduced as compared to those recorded when the mature heavy and mature light chains were combined. Despite the fact that trimeric QH0692 gp140 bound to both gHC/mLC and mHC/gLC, it was not able to mediate calcium mobilization through these two BCRs. This contrasts with the above-discussed observation made with trimeric SF162 gp140, which bound the mHC/gLC chimera and mediated calcium mobilization through the corresponding BCR. At this stage we can only speculate that the inability of the trimetric QH0692 gp140 to activate the mHC/gLC BCR is related to inefficient BCR cross-linking on the surface of B cells.

Although we examined diverse Envs from three different clades, potentially a much larger screen of Envs is required to identify those rare constructs that engage the germline BCRs of the type of antibodies we examined here. For instance, it may be that the development of such antibodies during natural HIV-1 infection depends on the emergence of particular Env clones in certain HIV-infected subjects; clones with particular structural characteristics that allow them to engage the germline BCR form of bNAb to the CD4-BS. In the context of HIV-1-infection, an alternative possibility is that the maturation of bNAb precursors was initiated in response to non-HIV antigens, and that the resultant maturation antibody intermediates were capable of recognizing bNAb epitopes on Env, as proposed for anti-gp41 antibodies [50]. We also note the possibility (not investigated here) that in the context of infection, the germline BCRs of these antibodies are initially engaged by membrane-anchored Env (for example, on the virion surface or on the surface of infected cells).

As mentioned above it is not possible to predict the exact amino acid composition of the VD and DJ joining regions in the true germline versions of the antibodies examined here. Therefore, the amino acids present in the mature antibodies in these two joining regions were left unchanged. One could argue therefore that these amino acids are not present in the ‘true’ germline BCRs of these antibodies, and that in the absence of other mutations in V and J regions, they play an inhibitory role in binding to certain recombinant Envs. Finally, we note that the results presented here were obtained with three NAbs whose epitopes are located within the same region of Env; the CD4-BS. It remains to be determined whether similar observations will be made with the germline versions of NAbs that bind to epitopes outside the CD4-BS. In this regard, it was reported that the germline versions of two MAbs, CH01 and CH04, which bind complex quaternary epitopes located outside the CD4-BS, can recognize certain recombinant Envs [51]. Similarly, germline versions of the anti-gp41 MAb 2F5 recognize certain forms of recombinant gp41 [52].

Overall our study provides new insights as to a potential reason why recombinant Env immunogens have failed to elicit anti-CD4-BS antibodies that display cross-neutralizing activities. Our results indicate that, as a first step in eliciting broadly neutralizing anti-CD4-BS antibodies by immunization, Env immunogens should be designed that engage the germline BCR versions of broadly neutralizing anti-CD4-BS antibodies.

## Materials and Methods

### Construction of plasmids expressing soluble b12 IgG variants

To express soluble b12 IgG variants, two separate pTT5-based vectors containing the b12 heavy and light chain variable and constant region open reading frames were used (provided by Dr. Pamela Bjorkman, California Institute of Technology, Pasadena, CA). The b12 heavy and light chain germline variable sequences from the original donor from which b12 was isolated [44] were used to replace the mature b12 variable regions in the pTT5 vectors using an *Eco*RI-*Not*I strategy for the heavy chain and *Eco*RI-*Eco*RI for the light chain.

Point mutations in the b12 germline heavy chain sequences were introduced using oligonucleotide mediated site directed mutagenesis (Stratagene Quick Change II system from Agilent Technologies, Santa Clara, California). For constructs bearing multiple point mutations, sequential rounds of site-directed mutagenesis were performed, with the exception of the residues at position 52 and 53 in the heavy chain, which were mutated in combination with a single set of mutagenic primers. Oligonucleotide primers used in this study are listed in **Table S1**.

Mature/germline heavy and light chain b12 chimeras were generated by co-expressing the plasmid encoding the germline heavy chain with the plasmid encoding the mature light chain (Chimera 1, also referred to as gHC/mLC), or by co-expressing the plasmid encoding for the mature heavy chain with the plasmid encoding the germline light chain (Chimera 2, also referred to as mHC/gLC). The germline sequences of NIH45-46 and 3BNC60 were previously reported [7].

### Plasmids expressing additional IgGs

Plasmids expressing the heavy and light chains of the mature and germline versions of NIH45-46 and 3BNC60 were kindly provided by Michel Nussenzweig (The Rockefeller University).

### Expression and purification of IgG

IgGs were produced by transient transfection of HEK 293e cells suspension cells, as previously described [53], [54] with a few modifications. Briefly, cells were suspended in FreeStyle 293 expression medium (Invitrogen, Carlsbad, CA) to a density of 20 million cells/mL. Heavy and light chain expressing pTT5 vectors were added to a final concentration of 6 µg/mL of each, immediately followed by the addition of linear PEI MAX (Polysciences Inc, Warrington, PA) to a final concentration of 24 µg/mL for 3 hours at 37°C. At this point the cell density was reduced to 1 million cells per mL in ExCell Vpro medium supplemented with 20 mM HEPES and 4 mM GlutaMax. Culture supernatants were harvested after 5 days and filtered through a 0.2 µM Millipore filter. Supernatants were diluted 1∶1 with PBS pH 7.4 and loaded on protein-A sepharose resin (GE healthcare) via gravity flow. IgG was eluted from the column using 0.1 M citrate, pH 2.5 into 1/10 volume of 1 M Tris pH 9.0.The purified IgG sample was buffer exchanged using 40 KDa MW cutoff Zeba desalting columns (Thermo Scientific). IgGs were quantitated by A_280_ and purity was verified by SDS-PAGE. IgG samples were aliquoted and stored at −20°C until use.

### Construction of BCR versions of b12 antibodies

To generate plasmids expressing the membrane-anchored version of the b12 IgGs (that is, with the transmembrane and cytoplasmic regions of human IgG1) the following strategy was used. The U-b12 plasmid [55] (provided by David Baltimore, CalTech) was used for these studies. The U-b12 plasmid expresses the mature heavy and light b12 IgG chains as a single polypeptide separated by a furin cleavage site and an F2A peptide sequence. Those regions were PCR amplified and replaced the b12 heavy chain insert in the pTT5-b12HC vector using the *Ecor*I and *Not*I sites. Thus, a pTT5 plasmid was generated that expresses both the heavy and light mature chains of IgG b12. The constant IgG splice variant cDNA (containing the two exons which encode the transmembrane and cytoplamic domains of human IgG) was synthesized by Genscript (Piscataway, NJ). That sequence replaced the soluble IgG heavy chain, the above pTT5 vector. The new vector, pTT5-mb12-BCR, expresses the membrane-anchored version of mature b12. The germline and the mature/germline chimeras were generated by replacing the mature b12 sequences by the appropriate germline VH and VL sequences.

### Recombinant HIV Envelope gp120 and gp140 proteins

Envelope proteins used in this study are listed in **Figure 2**. All recombinant gp120 proteins used in this study were obtained commercially (Immune Technologies, New York, New York), with the exception of SF162 gp120 which was produced in-house. D368R gp120 is derived from the SF162 gp120 by introducing an aspartic acid at position 368 within the CD4-BS. This mutation is known to abrogate the binding of CD4 to gp120, as well as the binding of most anti-CD4-BS antibodies [56], [57]. The Clade A: Q168a2, Q259d2, Q461e2, Q769h5; Clade B: SF162, SF162ΔV1, SF162ΔV2, SF162ΔV3, VC10042.05, VC10042.Y22.05.rm, VC10042.Y22.08 and VC10042.Y22.E1A; and Clade C: 140c, 327c, 405c, 426c, 459c, 706c, 756c, and 823c monomeric and/or trimeric gp140 proteins were expressed in mammalian cells and purified as we previously described by [17], [53], [54]. Clade B Envs JRCSF, SF162, ADA, JRFL, YU2 and HXBc2 were derived from viruses isolated during chronic infection. Clade C Envs Du151, Du422, CAP45, ZM249, ZM109F, ZM197M, ZM53M, ZM233M and Clade B Envs QH0692, AC10.0, PVO, TRO, RHPA, TRJO, WITO, REJO, THRO were derived from viruses isolated during the first 6 months of infection [10], [49], [58]. Clade A Envs Q168a2, Q259d2, Q461e2 and Q769h5 were derived from viruses isolated between 28 and 75 days post-infection [59] . The clade B Envs G4, C6a, F8, H5a and C4a were derived from a single HIV+ subject and isolated from 1–4 years post-infection. Clone G4 was isolated at year 1, F8 at 1.5 years, C6a at year 2, H5a at year 3.5 and C4a at 4 years post-infection (D. Malherbe et al, unpublished data). The clade C Envs 140c, 327c, 405c, 426c, 459c, 706c, 756c, and 823c were obtained during the first 3 months of infection from participants of the HVTN 503 clinical trial who became infected. Envs VC10042.05, VC10042.Y22.E1A, VC10042.Y22.08, VC10042.Y22.05.rm were derived from virus circulating in an elite neutralizer (VC10042) [3] at 22 years post-infection (DN Sather, submitted manuscript). All ‘in house’ produced Envs were expressed and purified as previously described [54], [60].

### ELISA

HIV gp120 and gp140 proteins were adsorbed onto 96-well Immulon 2HB-coated microtiter ELISA plates overnight at room temperature at a concentration of 0.5 µg/mL in 0.1 M NaHCO_3_ pH 9.5. Wells were washed 4 times in PBS + 0.02% Tween 20 (wash buffer) prior to blocking at 37°C for 2 hours with 300 µL per well of PBS containing 8% milk and 2% BSA. Plates were washed four times with wash buffer prior to addition of anti-Env MAbs in dilution buffer (PBS 8% milk 2% BSA 0.03% Tween 20). MAbs were added at a concentration of 30 µg/mL and three-fold serial titrations were performed (in duplicate), followed by a 1 hour incubation at 37°C. Wells were washed 4 times (PBS + 0.02% Tween 20) and HRP-conjugated goat anti-human IgG (Southern BioTech, Birmingham, Alabama) was added at a 1∶3000 dilution in dilution buffer for 1 hour at 37°C. At that point, the wells were washed 4 times with wash buffer and 50 µL of Ultra TMB substrate (Thermo Scientific/Pierce, Rockford Illinois) was added for three minutes at which point 50 µL of 1 N H_2_SO_4_ were added per well. The A_450_ of each well was read on a Versamax plate reader, and the analysis was performed using the Prism 5 package (Graphpad Software). Each experiment was independently replicated two to four times. The background absorbance (due to minimal non-specific binding of MAbs to wells) was calculated by taking the mean plus the standard deviation of the background absorbances against SF162 D368R gp120 of all MAbs tested. That value was determined to be equal to an A_450_ of 0.16 nm.

### Biolayer interferometry analysis of MAb-Env binding

The Octet KQe instrument (ForteBio, Inc, Menlo Park, CA) was used to perform biolayer interferometry (BLI) to detect the interaction of MAbs with soluble recombinant Env proteins.

(a) Antibody-capture method: Mature, germline and mature/germline chimeric b12 IgGs (at 15 µg/mL in PBS) were immobilized onto anti-human IgG FC capture (AHC) biosensors (Fortebio). The biosensors were then immersed into wells of a 96 well plate containing Env proteins at 1.02 µM concentration in 1× kinetics buffer (1× PBS, 0.01% BSA, 0.02%, Tween 20, and 0.005% NaN_3_) (association phase). The biosensors were subsequently immersed into wells containing only buffer (dissociation phase). Biosensors were regenerated using 0.1 M glycine (pH 1.7) followed by neutralization in buffer and reloading of IgG. The following method program was used for each sample tested: 1) Baseline measurement for 180 sec, 2) IgG loading for 300 sec, 3) Baseline measurement for 180 sec, 4) Association phase for 300 sec, 5) Dissociation phase for 600 sec. These steps were followed by regeneration for 15 sec and neutralization for 15 sec, repeated for 3 cycles.

(b): Antigen-capture method: Recombinant His-tagged antigens (at 20 µg/mL in PBS) (Immune Technologies were immobilized onto Ni-NTA biosensors (Fortebio). The biosensors were then immersed into wells of a 96 well plate containing mature, germline and mature/germline chimeric NIH45-46 and 3BNC60 IgGs at 100 nM concentration in 1× kinetics buffer (association phase). The biosensors were subsequently immersed into wells containing only buffer (dissociation phase). Biosensors were regenerated using three cycles of treatment with 0.1 M glycine (pH 1.7) followed by neutralization in 1× kinetics buffer followed by a treatment with 10 mM NiCl_2_. The following method program was used for each sample tested: 1) Baseline measurement for 300 sec, 2) Env loading for 300 sec, 3) Baseline measurement for 60 sec, 4) Association phase for 1200 sec, 5) Dissociation phase for 1200 sec. These steps were followed by regeneration for 15 sec and neutralization for 15 sec, repeated for 3 cycles and a final recharge step for 60 sec. Negative binding using the antigen capture method was defined as having a less than 0.2 nM shift.

*K*_a_, *k*_a_ and *k*_d_ determination was performed by antibody-capture BLI using 2-fold serial dilutions of recombinant Env over a range of concentrations roughly 10-fold below and 10-fold above the approximate *K*_a_ for each mAb tested. The reported *K*_a_, *k*_a_ and *k*_d_ values were determined by taking the mean of the *K*_a_, *k*_a_ and *k*_d_ values from all binding curves that matched the theoretical fit with an R^2^ value of ≥0.95.

### Viral neutralization assays

To test the neutralization potency of b12 IgG variants, inhibition of viral entry in luciferase reporter cells lines was performed as described previously [2], [3], [26], [61]. For CCR5-tropic viruses, the TZM-bl cell line was used as target, and for CXCR4-tropic viruses, the NIH-U87 cell line expressing CD4 and the CXCR4 co-receptor was used as target. The cells were maintained in DMEM, (Dubelco's Modified Eagle's Medium, Cellgrow) supplemented with 10% fetal bovine serum, 2 mM L-glutamine, 100 U/ml penicillin, and 100 µg/ml streptomycin. TZM-bl cells were plated at a density of 5×10^4^ cells per well and U87 cells at a density of 7×10^4^ per well 24 hours prior to addition of virus and antibody. Antibodies serially diluted in microtiter wells starting at a concentration of 20 µg/mL and a total volume of 30 µL per well. 30 µL of virus (previously determined to result in 1×10^5^ luciferase units was added to each well containing the titrated antibodies. Antibody-virus mixtures in duplicate wells were incubated at 37°C for 1.5 hours. During the last 30 minutes of the incubation, cells were treated at 37°C with 2 µg/mL of polybrene. The polybrene was then aspirated from the cells and replaced with 50 µL of antibody-pseudovirus mixture. Plates were incubated at 37°C for 72 hours, media was aspirated and cells were lysed to measure luciferase activity using Steady-Glo Luciferase Reagent (Promega, Madison, Wisconsin). The percent neutralization at each MAb concentration was determined as previously described [17], [61].

### B cell lines

The A20 mouse B cell lymphoma (ATCC # TIB-208) and the DG-75 human Burkitt's lymphoma (ATCC # CRL-2625) cell lines were used for these assays. A20 cells were maintained in RPMI-1640 supplemented with 10% FBS and 0.05 mM β-mercaptoethanol and the DG-75 cells were maintained in RPMI-1640 supplemented with 10% FBS. DG-75 cells express human IgM on their surface, while A20 cells express mouse IgG on their surface.

### Transfection of B cell lines with exogenous BCRs

Both A20 and DG-75 B cell lines were used for the cell surface expression of human IgG BCRs. 2×10^6^ cells were re-suspended in 100 µl of cell line nucleofector solution V (Lonza, Cologne Germany) containing 5 µg of the appropriate pTT5-BCR plasmid. Transfections of A20 and DG-75 cells were performed using Amaxa Nucleofector II, programs L-013 and O-006, respectively (Lonza, Cologne Germany). Surface staining of exogenous human BCRs was performed using allophycocyanin (APC) conjugated mouse monoclonal anti-human IgG (BD Pharmingen Cat# 550931) at a 1/10 dilution. Exogenous human BCR cross-linking on the surface of B cells was achieved with the use of the goat anti-human IgG F(ab′)_2_ fragment (20 µg/ml; Jackson Immunoresearch Cat. # 309-005-006).

### Analysis of intracellular calcium mobilization

24 h post-transfection with plasmids expressing human BCRs, A20 or DG-75 cells were loaded with Fluo-4 Direct calcium indicator (Invitrogen, Carlsbad CA) in RPMI-1640 medium containing 10% FBS at 37°C for 1 h. The cells were then washed twice with 5 ml in RPMI-1640 containing 10% FBS, resuspended at ∼1 million cells/ml in RPMI-1640 and subjected to analysis of calcium flux at a medium flow rate on an LSR II cytometer (Beckton Dickinson).

(a) Defining the functionalities of the exogenous BCRs: Minimum levels of background fluorescence (Min_FL_) were determined by averaging the background Fluo-4 fluoresence in cells for 30 s. After determining Min_FL_, exogenous BCR cross-linking on the surface of transfected cells was performed in mouse A20 cells by the addition of goat anti-human IgG (H+L) F(ab′)_2_ to a concentration of 20 µg/ml. Cell-associated changes in Fluo-4 fluorescence were further monitored for 210 seconds. At that time, ionomycin was added to a final concentration of 6.5 nM for 60 s. Maximum Fluo-4 fluorescence (Max_FL_) recorded following the addition of ionomycin was determined by averaging changes in Fluo-4 fluorescence recorded between 290 s and 300 s.

(b) Recombinant soluble Env-mediated BCR activation: The ability of recombinant soluble Env proteins to stimulate calcium flux in DG-75 cells expressing the exogenous BCRs. Once the Min_FL_ was determined as discussed above, soluble recombinant Env proteins were added to a final concentration of 30 µg/ml. Changes in Fluo-4 fluorescence associated with cells expressing the exogenous BCRs (APC positive cells) were further monitored for 210 seconds, at which point ionomycin was added and the Max_FL_ was determined.

For both (a) and (b) the percent of maximum Fluo-4 fluorescence at each time point t was determined using the formula: (Fluorescence at t-Min_FL_)/(Max_FL_-Min_FL_)×100. This analysis was performed on both the BCR positive (anti-IgG-APC positive) and BCR negative cells (anti-IgG-APC negative) simultaneously. The background Fluo-4 fluorescence signal from the BCR negative cells was subtracted from that of the BCR positive population at each time point.

## Supporting Information

Figure S1 **Amino acid alignment of b12 mature and germline heavy and light chain variable regions for NIH 46-46 (A) and 3BNC60 (B).** The framework (FR) and complementary determining regions (CDR) are outlined, and the D- and J- gene segments are boxed. The complementarity determining (CDR) and framework (FW) regions were determined using the IMGT/V-Quest tool (www.imgt.org). Amino acid numbering is based on the Kabat numbering system.
(TIF)

Figure S2 **Schematic representation of mature, germline and chimeric b12 antibodies.**
(TIF)

Table S1 **Oligonucleotides used in this study.**
(DOCX)
