# Correction: Recombinant HIV Envelope Proteins Fail to Engage Germline Versions of Anti-CD4bs bNAbs

**DOI:** 10.1371/annotation/ed7c0148-97eb-4416-824d-6e6d1aaeceef

**Published:** 2013-07-05

**Authors:** Sam Hoot, Andrew T. McGuire, Kristen W. Cohen, Roland K. Strong, Lars Hangartner, Florian Klein, Ron Diskin, Johannes F. Scheid, D. Noah Sather, Dennis R. Burton, Leonidas Stamatatos

In the Funding section, the grant number from the funder NIAID/HIVRAD was listed incorrectly. The correct grant number is: P01AI094419.

