# Supplementary figures and images for: Recombinant HIV Envelope Proteins Fail to Engage Germline Versions of Anti-CD4bs bNAbs
Source: PLoS Pathog. 2013 Jan 3;9(1):e1003106. doi: 10.1371/journal.ppat.1003106 (PMC3536657; doi:10.1371/journal.ppat.1003106)

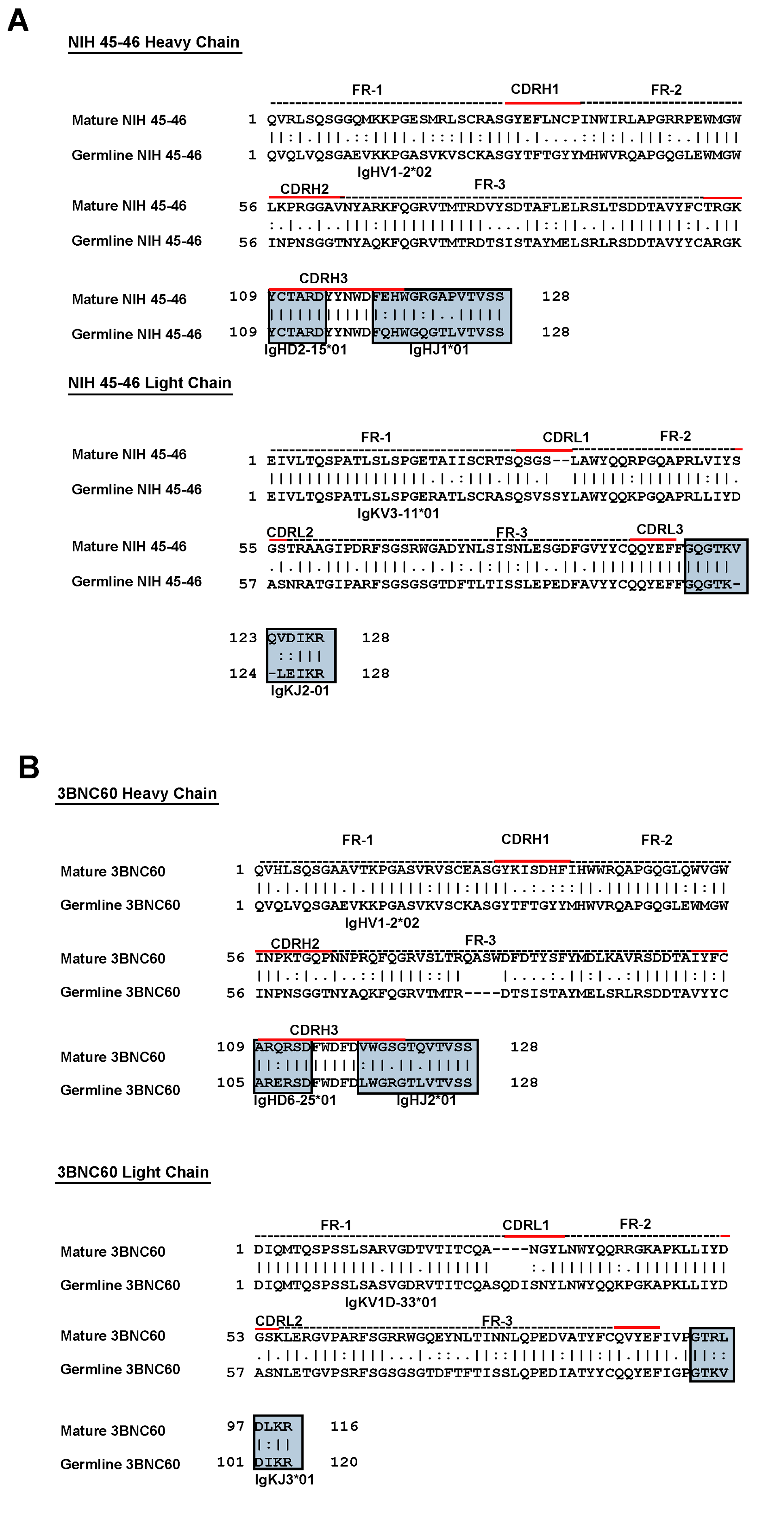

Supplement: Figure S1 — Amino acid alignment of b12 mature and germline heavy and light chain variable regions for NIH 46-46 (A) and 3BNC60 (B). The framework (FR) and complementary determining regions (CDR) are outlined, and the D- and J- gene segments are boxed. The complementarity determining (CDR) and framework (FW) regions were determined using the IMGT/V-Quest tool (www.imgt.org). Amino acid numbering is based on the Kabat numbering system. (TIF) [file ppat.1003106.s001.tif]

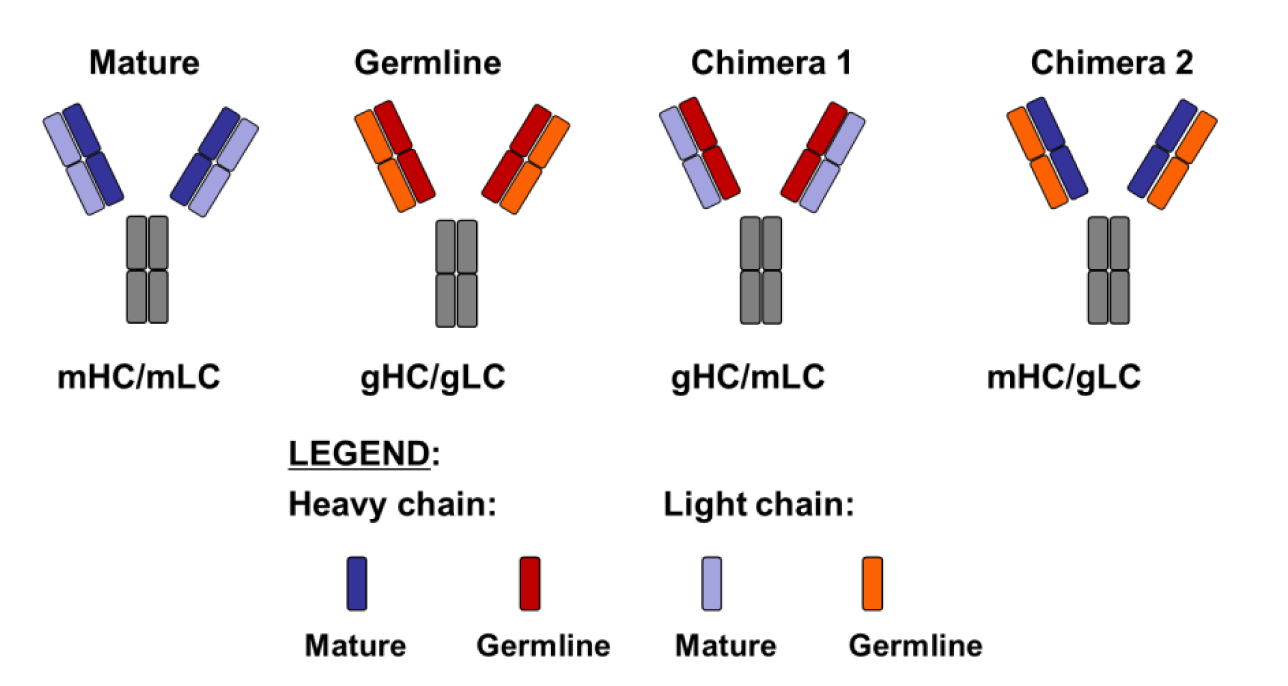

Supplement: Figure S2 — Schematic representation of mature, germline and chimeric b12 antibodies. (TIF) [file ppat.1003106.s002.tif]
