# Supplementary material for: Recombinant HIV Envelope Proteins Fail to Engage Germline Versions of Anti-CD4bs bNAbs
Source: PLoS Pathog. 2013 Jan 3;9(1):e1003106. doi: 10.1371/journal.ppat.1003106 (PMC3536657; doi:10.1371/journal.ppat.1003106)
Supplement: Table S1 — Oligonucleotides used in this study. (DOCX) [file ppat.1003106.s003.docx]

**Table S1: Oligonucleotides used in this study.**

| **OLIGO NAME** | **Mutation** | **SEQUENCE 5'-3'** |
| --- | --- | --- |
| b12 germHC pTT5_*Eco*RI | N/A | GAATTCGCCGCCACCATGGAATGGAGCTGGGTC |
| b12 germHC pTT5rev_*Not*I | N/A | GCGGCCGCTCATTTACCCGGAGACAGGGAGAG |
| b12g S31N | CDRH1 | GATACACCTTCACTAACTATGCTATGCATTGGGTGC |
| b12g S31N rev | CDRH1 | GCACCCAATGCATAGCATAGTTAGTGAAGGTGTATC |
| b12g A52P G53Y | CDRH2 | GGGATGGATCAACCCTTACAATGGTAACACAAAATATTC |
| b12g A52P G53Y rev | CDRH2 | GAATATTTTGTGTTACCATTGTAAGGGTTGATCCATCCC |
| b12g G100W | CDRH3 | GTGGGGCCATATTGTTGGGGTGACTCTCCCCAGGAC |
| b12g G100W rev | CDRH3 | GTCCTGGGGAGAGTCACCCCAACAATATGGCCCCAC |
